# Signal of venous thromboembolism with ethinyl estradiol vs natural estrogens in oral contraceptives: analysis of the EudraVigilance and Food and Drug Administration Adverse Events Reporting System databases

**DOI:** 10.1016/j.rpth.2026.107005

**Published:** 2026-09-30

**Authors:** Lucie Raskin, Marie Didembourg, Médéa Locquet, Laure Morimont, Charlotte Beaudart, Jean-Michel Dogné, Jonathan Douxfils

**Affiliations:** 1 Research Unit in Clinical Pharmacology and Toxicology, Namur Research Institute for Life Sciences (NARILIS), Faculty of Medicine, University of Namur, Namur, Belgium; 2 Public Health Aging Research & Epidemiology (PHARE) Group, Research Unit in Clinical Pharmacology and Toxicology, Namur Research Institute for Life Sciences (NARILIS), Faculty of Medicine, University of Namur, Namur, Belgium; 3 Department of Biological Hematology, CHU Clermont-Ferrand, Hôpital Estaing, Clermont-Ferrand, France

**Keywords:** estetrol, estradiol, oral contraceptives, pharmacovigilance, venous thrombosis

## Abstract

**Background:**

Disproportionality analyses in the Food and Drug Administration Adverse Events Reporting System (FAERS) and EudraVigilance reported lower reporting of venous thromboembolism (VTE) with natural estrogen–based combined oral contraceptives (COCs) than with ethinylestradiol/levonorgestrel. However, using ethinylestradiol/levonorgestrel as the reference showed reduced reporting only for natural estrogen–based products. We therefore reanalyzed both databases separately, using natural estrogen–based COCs as the reference.

**Methods:**

Previously extracted individual case safety report datasets and published disproportionality analyses were re-examined, using natural estrogen–based contraceptives as the comparator. Proportional reporting ratios (PRRs) were recalculated separately for each database. COCs were categorized as ethinylestradiol-based or natural estrogen–based formulations. Drospirenone-only pills were included for comparison. Analyses used pooled natural estrogen contraceptives, estetrol/drospirenone, or estradiol/dienogest as reference groups. Disproportionality was defined as PRR >2, at least 3 cases, and χ^2^ ≥ 4.

**Discussion:**

Consistent patterns were observed in EudraVigilance and FAERS. Compared with natural estrogen–based COCs, ethinylestradiol-based COCs showed a disproportionate reporting of VTE (EudraVigilance: PRR, 5.10 [95% CI, 4.68-5.56]; FAERS: PRR, 4.21 [95% CI, 3.49-5.08]). In EudraVigilance, when using estetrol/drospirenone as the reference, all ethinylestradiol-based formulations showed disproportionality according to the conventional PRR threshold. A similar trend was noted in FAERS, though not statistically significant, with the drospirenone-only formulation comparable to estetrol/drospirenone.

**Conclusion:**

This analysis revealed a consistent and disproportionate reporting of VTE associated with ethinylestradiol-based COCs compared with natural estrogen–based alternatives across 2 pharmacovigilance databases. These results, together with prior biological and observational research, support further reevaluation of COC options given the estrogenic component’s potential influence on VTE risk.

## Essentials

• Blood clot risk may differ by the estrogen used in combined birth control pills.
• We compared safety reports from European and US databases.
• Pills with synthetic estrogen had over 4 times the share of blood clot reports.
• Multiple levels of evidence support a favorable profile of natural estrogen pills.

## 1 Introduction

Combined oral contraceptives (COCs), which contain an estrogen and a progestin, are a known risk factor for venous thromboembolism (VTE) [1]. Despite various efforts to reduce the risk, VTE related to COCs containing ethinylestradiol (EE) remains a notable public health issue, with an estimated incidence of 5 to 12 cases per 10,000 woman-years depending on the COC used. Over 22,000 and 9000 COC-associated VTE cases occur annually in Europe and the United States, respectively [2]. Because of concerns about VTE risk, regulators such as the European Medicines Agency (EMA) and the Food and Drug Administration initiated a re-evaluation of COCs approximately 10 years ago and found that the benefits of COCs still outweigh the risks [3], [4]. The risk of VTE depends not only on the E dose but also on the type of associated progestin [2]. Today, formulations such as EE/levonorgestrel (EE/LNG) and EE/norgestimate (EE/NGM) are preferred in several countries because their thrombotic risk profiles are well documented. However, improving the safety profile of COCs calls for new strategies [2].

Multiple levels of evidence now indicate that the type of estrogen, not only its dose, plays an important role [5]. Although most COCs traditionally contain EE, a contraceptive class based on natural estrogens (eg, estradiol [E2] and estetrol [E4]) was introduced in the late 2000s and seemed to have a neutral profile on hemostasis [6]. This evidence comes from biological studies [7], [8], [9], [10], [11] and observational cohorts (eg, INAS-SCORE [International Active Surveillance Study—Safety of Contraceptives: Role of Estrogens. It evaluated estradiol valerate/dienogest], PRO-E2 [Prospective Controlled Cohort Study on the Safety of a Monophasic Oral Contraceptive Containing Nomegestrol Acetate (2.5 mg) and 17β-Estradiol (1.5 mg)], and Nordic registries) [12], [13], [14] and suggests that COCs containing natural estrogens, such as E2, E2 valerate, or E4, may be associated with a lower risk of VTE compared with EE-based combinations. Moreover, a recent meta-analysis of regulatory-imposed postauthorization safety studies (PASS) with natural estrogens reported that EE/LNG is associated with a 95% increase in the risk of VTE compared with E2-based pills (adjusted hazard ratio [HR], 1.95; 95% CI, 1.11-3.41) [15]. In addition, a pharmacodynamic prediction model, based on an EMA-recommended biomarker for the hemostatic evaluation of steroid contraceptives [16], [17], strengthens this evidence by estimating a significantly lower risk of VTE with natural estrogens, including E2 and E4, compared with EE-based pills [18].

Recently, 2 disproportionality analyses, conducted independently in the Food and Drug Administration Adverse Events Reporting System (FAERS) [19] and EudraVigilance [20], showed lower reporting of VTE with natural estrogen–based contraceptives than with EE-containing products. However, because EE/LNG or EE-based contraceptives are used as reference groups, these analyses may highlight the lower reporting for natural estrogens but cannot formally establish a pharmacovigilance signal for EE-based contraceptives.

Therefore, this descriptive analysis aims to re-examine the FAERS and EudraVigilance results, using natural estrogen–based contraceptives as the reference group, to investigate whether the previously observed reduction in VTE reporting with natural estrogens can be used to define a pharmacovigilance signal for excess reporting of VTE events with EE-containing pills.

## 2 Methods

In both disproportionality studies, the same methodology was applied [19], [20]. All VTE events were defined using the same criteria and coding framework, based on the Medical Dictionary for Regulatory Activities. Furthermore, both analyses were restricted to individual case safety reports within the COC therapeutic class, thereby reducing confounding by indication and limiting background reporting noise. The strong methodological consistency between the 2 studies enabled a direct comparison of reporting patterns across the European and US pharmacovigilance databases, although differences in geographic coverage, reporting practices, healthcare systems, prescribing patterns, and pharmacovigilance regulations may influence reporting behavior and the resulting disproportionality estimates [21].

Using natural estrogen–containing COCs as a common reference point, we investigated whether a pharmacovigilance signal emerges with EE-based COCs across both databases. First, all EE-based COCs were grouped together and compared with COCs containing natural estrogens. E2/dienogest (DNG) and E4/drospirenone (DRSP) were considered separately as a single reference group for natural estrogens because both formulations have individually demonstrated limited effects on key hemostatic biomarkers associated with VTE risk, including activated protein C resistance and thrombin generation parameters [11]. Secondly, E2/DNG and E4/DRSP each served as new reference points and were assessed with every COC formulation. Since E2/nomegestrol acetate (NOMAC), EE/chlormadinone acetate, EE/cyproterone acetate, EE/gestodene, and EE/DNG were reported only in the EudraVigilance database, they were excluded from this descriptive analysis to maintain consistency across databases. DRSP, a progestin-only pill, was included for comparison. The proportionality reporting ratio (PRR) was computed as (A/[A+B])/(C/[C+D]), where A is the number of thrombotic events for the drug under evaluation, and B is the number of nonthrombotic events for the drug under evaluation, whereas C is the number of thrombotic events for the comparator, and D is the number of nonthrombotic events for the comparator. This analysis also defined pharmacovigilance signals based on Evans et al.’s [22] criteria, which include a PRR of at least 2, at least 3 reported cases, and a χ^2^ value of 4 or higher.

## 3 Discussion

Both pharmacovigilance databases consistently showed that EE-based COCs were associated with a higher proportion of VTE reports than natural estrogen–based COCs (Figure 1). This finding supports the evidence that COCs containing natural estrogen may have a more favorable VTE risk profile than EE-based COCs. Indeed, compared with natural estrogen–based COCs, EE-based COCs showed significantly higher PRRs (EudraVigilance: PRR, 5.10 [95% CI, 4.68-5.56], χ^2^ = 3625, *P* < .001; FAERS: PRR, 4.21 [95% CI, 3.49-5.08], χ^2^ = 295.72, *P* < .001), indicating a pharmacovigilance signal (Figure 1). The DRSP-only pill showed lower to similar PRRs compared with natural estrogens (EudraVigilance: PRR, 0.56 [95% CI, 0.45-0.70], χ^2^ = 28.872, *P* < .001; FAERS: PRR, 1.04 [95% CI, 0.82-1.33], χ^2^ = 0.083914, *P* = .77) (Figure 1). This is consistent with previous pharmacodynamic data [23], [24]. The similar patterns seen in the FAERS and EudraVigilance databases reinforce the robustness of the findings, despite the inherent differences in their reporting systems [21]. Using E2/DNG or E4/DRSP alone as the reference is also informative: it shows that all EE-based formulations have a disproportionality signal (PRR >2; χ^2^ > 4) in the EudraVigilance database. Conversely, the DRSP-only pill had a similar or lower PRR, whereas EE/DRSP exhibited the highest PRR, suggesting that the estrogenic component may contribute to differences in VTE reporting [5]. A similar pattern was observed in the FAERS database, although it did not reach statistical significance.

**Figure 1:**
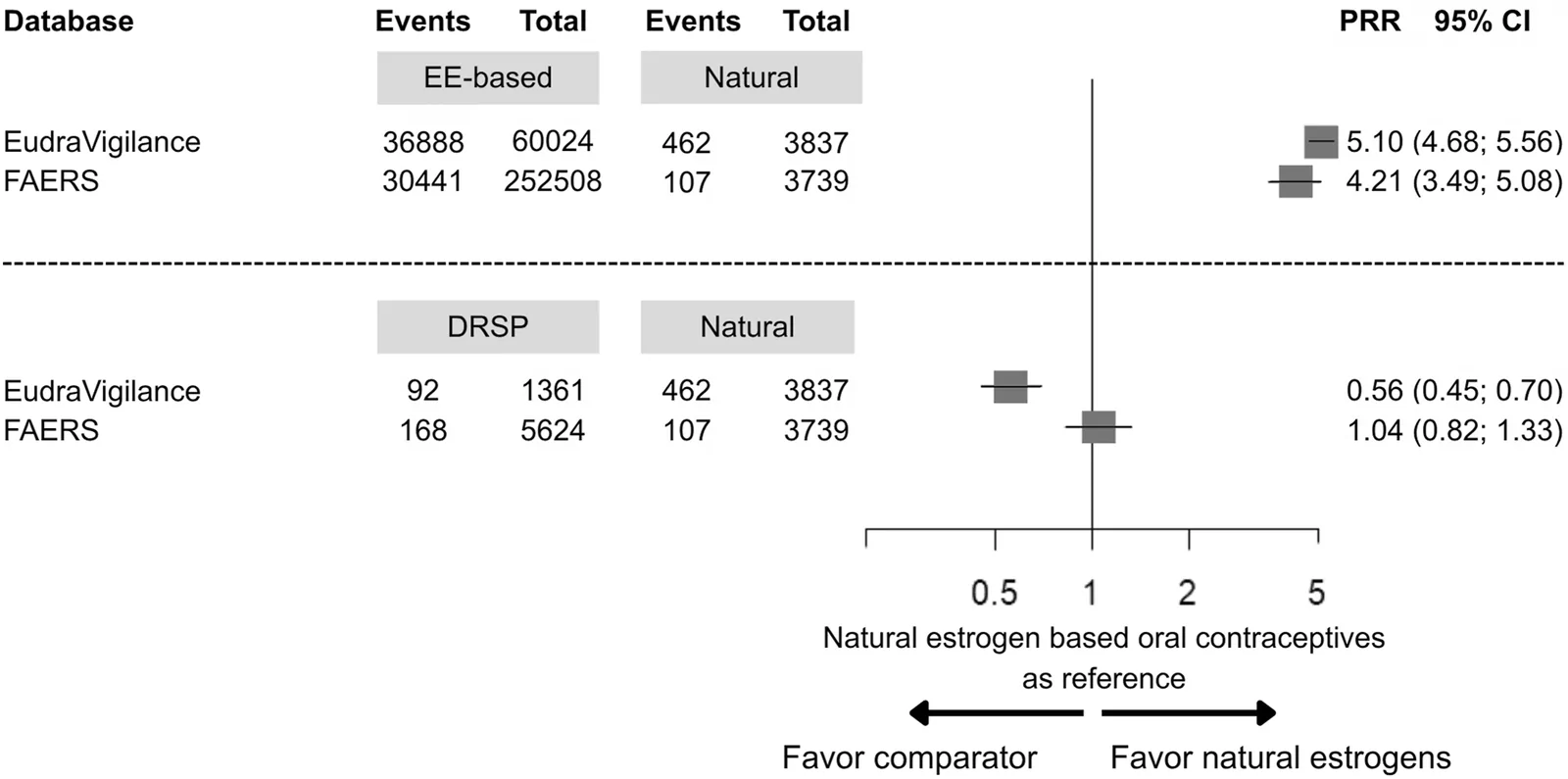
Descriptive analysis of EudraVigilance and FAERS data with natural estrogen–based COCs (E2/DNG and E4/DRSP) as the reference. The PRR compares the proportion of specific adverse events (eg, thrombotic adverse events) for a drug (A/[A+B]) with that for a comparator (C/[C+D]), where A is the number of thrombotic events, and B is the number of nonthrombotic events for the drug under evaluation; C is the number of thrombotic events, and D is the number of nonthrombotic events of the comparator. COC, combined oral contraceptive; DRSP, drospirenone-only pill; E2, estradiol; E4, estetrol; EE-based, all ethinylestradiol-based COCs (EE/LNG, EE/NGM, and EE/DSG; EE/DRSP); FAERS, Food and Drug Administration Adverse Events Reporting System; Natural, natural estrogen–based COCs (E2/DNG and E4/DRSP); PRR, proportional reporting ratio.

These observations are consistent with biological evidence showing that E2- and E4-containing contraceptives have a lower impact on hemostatic parameters, including activated protein C resistance and thrombin generation, than EE-based formulations [7], [8], [9], [10], [11], [25]. Indeed, higher normalized activated protein C sensitivity ratio values have been observed with EE/LNG compared with COCs containing natural estrogens in several independent studies [6], [7], [8], [9], [11], [18]. Moreover, a recent meta-analysis of observational studies, including PASS required by the EMA (eg, PRO-E2 and INAS-SCORE) [12], [13], demonstrated a significant 51% increase in VTE risk among EE-based COC users vs E2-based COC users (Peto odds ratio, 1.51 [95% CI, 1.17-1.95]). After adjusting for confounding factors according to EMA PASS protocols, the pooled HR even revealed a significant 95% increase in VTE risk among EE/LNG users compared with E2-based COC users (HR, 1.95 [95% CI, 1.11-3.41]) [15].

Although disproportionality analyses cannot establish causality or quantify absolute risk, they provide an additional pillar of evidence supporting COCs containing natural estrogens by capturing real-world patterns across large spontaneous reporting systems. They enable early detection of safety signals through large, spontaneous reporting databases that reflect actual use. Although observational studies are crucial for validating and understanding risks, they are often time-consuming and necessitate extensive data collection and follow-up procedures, delaying the generation of evidence. Disproportionality analyses, however, enable rapid identification of safety signals across populations, facilitating early prioritization of potential safety issues [26]. Based on 2 large, independent real-world databases from the United States and Europe, this analysis complements biological and observational evidence by providing consistent pharmacovigilance signals that are compatible with previously reported differences in thromboembolic risk between EE-containing and natural estrogen–based contraceptives.

Taken together, pharmacovigilance signals, observational evidence, and biological data highlight the need for a careful re-examination of VTE risk differences between EE-based COCs and natural estrogen alternatives. Such re-evaluation should be conducted at the international level within frameworks such as the Data Analysis and Real World Interrogation Network (DARWIN EU), which enables structured observational studies using continuous real-world healthcare databases [27]. The Expert Scientific Committee on Natural Estrogens and Council is already advising a reassessment of the current guidelines to consider a shift in estrogen choices for COCs [28].

In addition to their potentially lower thrombotic risk, natural estrogens are associated with progestins that exhibit weak androgenic or antiandrogenic properties, such as DNG, NOMAC, and DRSP. DRSP, DNG, and NOMAC were designed to bind more specifically to progestogen receptors and, to a lesser extent, to other steroid receptors, thereby reducing undesirable effects such as acne, hirsutism, and fluid retention [29]. However, when combined with EE, these progestins have been associated with higher VTE reporting, which may have affected prescribing patterns and contributed to a more cautious perception of their safety. Although safety remains a primary concern, quality-of-life aspects are also important, as they influence contraceptive selection and, consequently, adherence.

As with all spontaneous reporting analyses, this investigation has limitations. The lack of adjustment for individual risk factors such as age, obesity, smoking, thrombophilia, and detailed estrogen/progestin exposure is a well-known limitation of spontaneous reporting databases. Because individual-level data are unavailable, differences in baseline risk profiles between users of different COCs cannot be adequately assessed or controlled for. The potential importance of these factors is illustrated by the INAS-SCORE and PRO-E2 observational studies, in which users of natural estrogen–containing COCs appeared to have a less favorable baseline risk profile than users of EE-based COCs, including differences in age and other cardiovascular risk factors [12], [13]. Such differences are consistent with channeling bias, whereby women perceived to be at higher baseline risk may preferentially receive contraceptive formulations with natural estrogens. Notably, adjustment for baseline characteristics shifted the comparative risk estimates further in favor of natural estrogen–containing COCs, indicating that baseline differences may influence the crude estimates [15], [30]. Additional limitations should be considered when interpreting these findings. Contraceptives included in this analysis have been marketed for different lengths of time. Newly introduced products, such as E4/DRSP, may be subject to the Weber effect, whereas reporting patterns for EE-containing contraceptives may be influenced by longstanding awareness of thrombotic risks, regulatory communications, scientific publications, and media attention, particularly for EE/DRSP formulation. Interestingly, such a pattern was not apparent for E4/DRSP, despite its more recent introduction compared with EE-containing formulations. Nevertheless, for the EudraVigilance database, PRRs have remained stable over the last decade for E2-based pills compared with EE/LNG, indicating that the main results are not driven by excess reporting during a specific timeframe [20]. Despite these limitations, the overall direction of the findings was generally similar across databases, providing some reassurance regarding the robustness of the observed trends (Figures 2 and 3).

**Figure 2:**
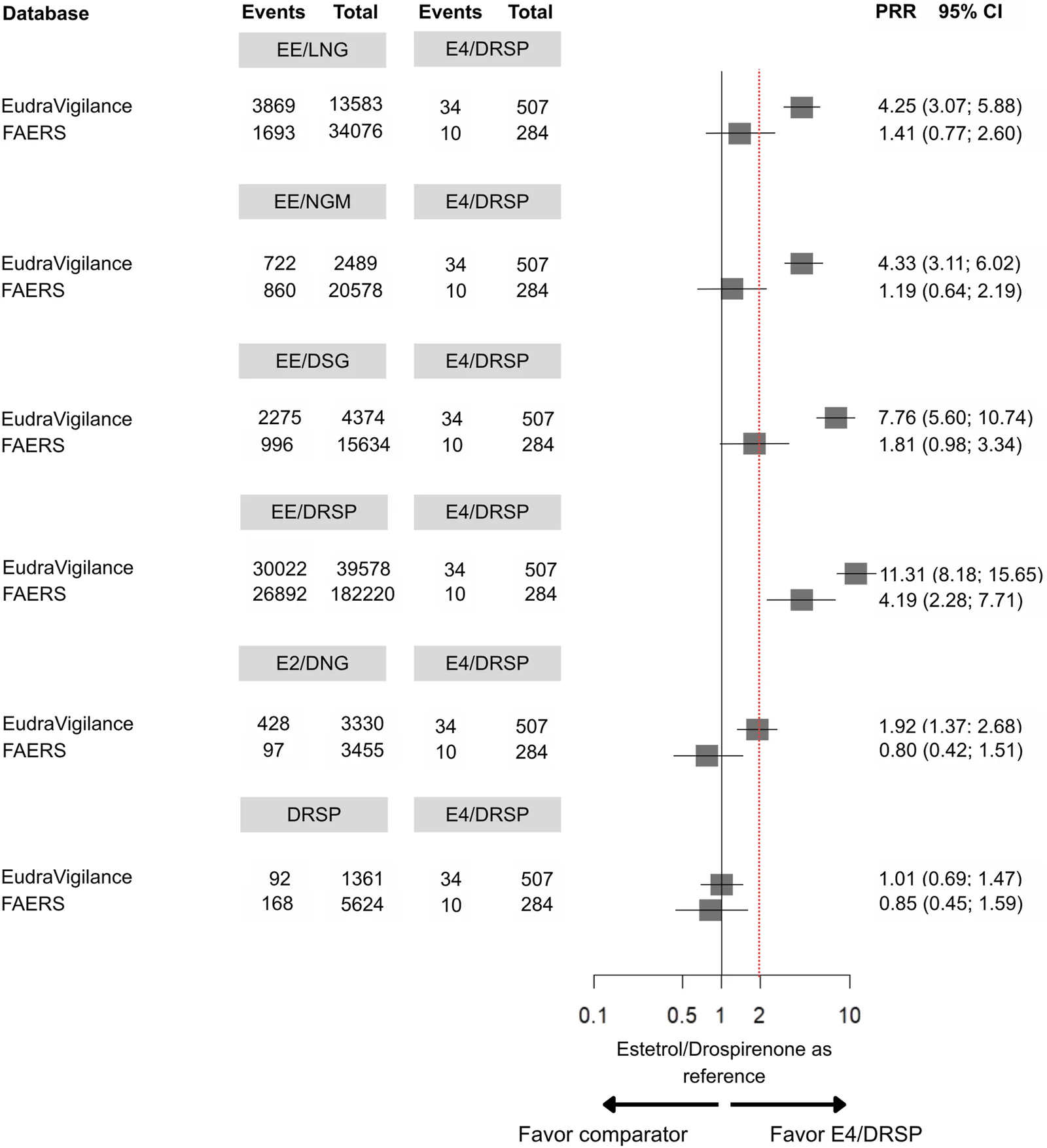
Descriptive analysis of EudraVigilance and FAERS data with E4/DRSP as the reference. The PRR compares the proportion of specific adverse events (eg, thrombotic adverse events) for a drug (A/[A+B]) with that for a comparator (C/[C+D]), where A is the number of thrombotic events, and B is the number of nonthrombotic events for the drug under evaluation; C is the number of thrombotic events, and D is the number of nonthrombotic events of the comparator. DRSP, drospirenone; E2/DNG, estradiol (valerate)/dienogest; E4/DRSP, estetrol/drospirenone; EE/DRSP, ethinylestradiol/drospirenone; EE/DSG, ethinylestradiol/desogestrel; EE/LNG, ethinylestradiol/levonorgestrel; EE/NGM, ethinylestradiol/norgestimate; FAERS, Food and Drug Administration Adverse Events Reporting System; PRR, proportional reporting ratio.

**Figure 3:**
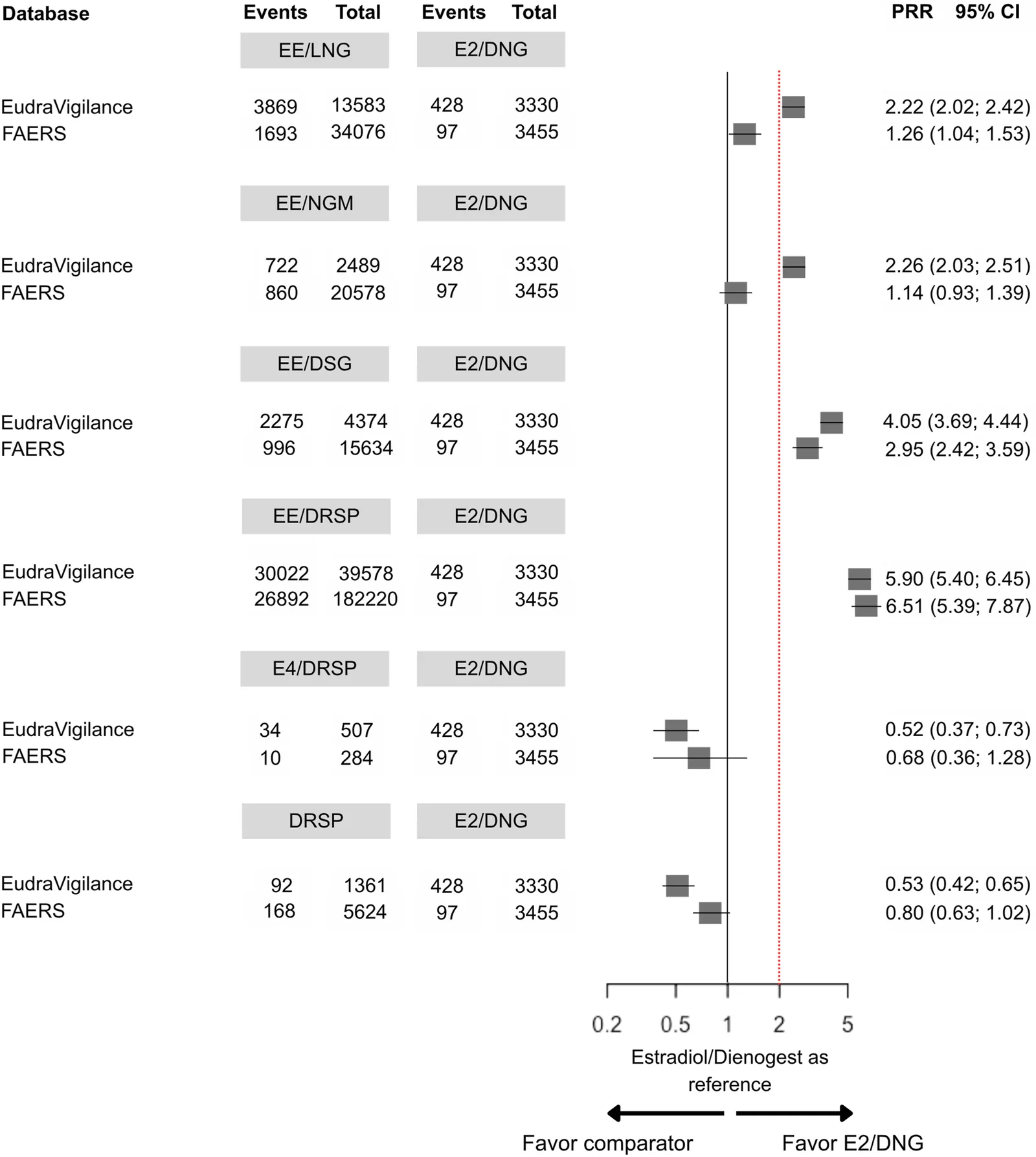
Descriptive analysis of EudraVigilance and FAERS data with E2/DNG as the reference. The PRR compares the proportion of specific adverse events (eg, thrombotic adverse events) for a drug (A/[A+B]) with that for a comparator (C/[C+D]), where A is the number of thrombotic events, and B is the number of nonthrombotic events for the drug under evaluation; C is the number of thrombotic events, and D is the number of nonthrombotic events of the comparator. DRSP, drospirenone; E2/DNG, estradiol (valerate)/dienogest; E4/DRSP, estetrol/drospirenone; EE/DRSP, ethinylestradiol/drospirenone; EE/DSG, ethinylestradiol/desogestrel; EE/LNG, ethinylestradiol/levonorgestrel; EE/NGM, ethinylestradiol/norgestimate; FAERS, Food and Drug Administration Adverse Events Reporting System; PRR, proportional reporting ratio.

In conclusion, this descriptive reanalysis of 2 large independent databases, FAERS and EudraVigilance, suggests a pharmacovigilance signal for VTE reports associated with EE-based COCs compared with natural estrogen formulations. These pharmacovigilance signals warrant further confirmation through appropriately designed observational studies before any regulatory or clinical conclusions can be drawn.
